# Kā-HOLO Project: a protocol for a randomized controlled trial of a native cultural dance program for cardiovascular disease prevention in Native Hawaiians

**DOI:** 10.1186/s12889-017-4246-3

**Published:** 2017-04-17

**Authors:** Joseph Keawe‘aimoku Kaholokula, Mele A. Look, Thomas A. Wills, Māpuana de Silva, Tricia Mabellos, Todd B. Seto, Hyeong Jun Ahn, Ka‘imi A. Sinclair, Dedra Buchwald, on behalf of the Kā-HOLO Project

**Affiliations:** 1 Department of Native Hawaiian Health, John A. Burns School of Medicine, University of Hawai‘i at Mānoa, Honolulu, USA; 2 University of Hawai‘i Cancer Center, Honolulu, USA; 3 Hālau Mōhala ‘Ilima, Kailua, USA; 4 Department of Complementary and Integrative Medicine, John A. Burns School of Medicine, University of Hawai‘i at Mānoa, Honolulu, USA; 5 Initiative for Research and Education to Advance Community Health (IREACH), Washington State University, Seattle, USA

**Keywords:** Hypertension, Cardiovascular disease, Cerebrovascular disease, Native Hawaiians, Randomized controlled trial

## Abstract

**Background:**

As a major risk factor for cardiovascular and cerebrovascular disease (CVD), hypertension affects 33% of U.S. adults. Relative to other US races and ethnicities, Native Hawaiians have a high prevalence of hypertension and are 3 to 4 times more likely to have CVD. Effective, culturally-relevant interventions are needed to address CVD risk in this population. Investigators of the Kā-HOLO Project developed a study design to test the efficacy of an intervention that uses hula, a traditional Hawaiian dance, to increase physical activity and reduce CVD risk.

**Methods:**

A 2-arm randomized controlled trial with a wait-list control design will be implemented to test a 6-month intervention based on hula to manage blood pressure and reduce CVD risk in 250 adult Native Hawaiians with diagnosed hypertension. Half of the sample will be randomized to each arm, stratified across multiple study sites. Primary outcomes are reduction in systolic blood pressure and improvement in CVD risk as measured by the Framingham Risk Score. Other psychosocial and sociocultural measures will be included to determine mediators of intervention effects on primary outcomes. Assessments will be conducted at baseline, 3 months, and 6 months for all participants, and at 12 months for intervention participants only.

**Discussion:**

This trial will elucidate the efficacy of a novel hypertension management program designed to reduce CVD risk in an indigenous population by using a cultural dance form as its physical activity component. The results of this culturally-based intervention will have implications for other indigenous populations globally and will offer a sustainable, culturally-relevant means of addressing CVD disparities.

**Trial registration:**

ClinicalTrials.gov: NCT02620709, registration date November 23, 2015.

## Background

Hypertension (HTN), defined as systolic blood pressure (SBP) ≥140 mmHg or diastolic blood pressure (DBP) ≥90 mmHg, is a serious public health concern in the U.S., affecting 33% of adults [1]. It is a major risk factor for cardiovascular and cerebrovascular diseases (CVD) such as coronary heart disease and stroke [2]. Native Hawaiians bear a disproportionate burden of CVD risk relative to the U.S. general population and individual racial and ethnic minority groups [3]. In Hawai‘i, the prevalence of HTN in Native Hawaiians is 48%, compared to 23% in Whites, 38% in Japanese Americans, and 30% in Filipino Americans [4–7]. Native Hawaiians are 3 times more likely to suffer heart disease and 4 times more likely to suffer stroke than Whites, and these conditions develop an average of 10 years earlier than in Whites [7, 8]. They are also 68% and 20% more likely to die of coronary heart disease and stroke, respectively, than the general population [9], but they are less likely to receive adequate HTN treatment [6, 10]. Thus, it is imperative that effective HTN interventions be implemented to prevent CVD in this population.

Native Hawaiians are descendants of the original inhabitants of the Hawaiian Islands, and in many respects their history parallels those of American Indians and Alaska Natives [11]. Before Western contact, Native Hawaiians were a healthy and robust people with a rich cultural tradition. After contact with Western powers, including the U.S., infectious diseases decimated the Native Hawaiian population. Large numbers were displaced from their ancestral lands; forced to abandon their native language, customs, and beliefs; marginalized through legislative acts and compulsory assimilation policies [12]; and subjected to discrimination in housing, education, employment, and health care [13]. The consequences of this historical trauma and institutionalized discrimination include high rates of CVD and other chronic illnesses [3, 14], which have resulted, in part, from cultural disruptions that altered traditional patterns of diet and physical activity [12].

Many contemporary Native Hawaiians report that they are alienated from and discriminated against by the Western-based health system, and that they lack access to culturally acceptable ways to manage their health [15, 16]. They believe that their cultural values and practices are often devalued by Western medicine, leading to mistrust and reluctance in seeking care from Western-trained medical providers [15–17]. Against this background, new health promotion strategies are needed in the form of programs supported by Native Hawaiian values and practices that reinforce rather than discard their preferred modes of living.

### Hula as a culturally appropriate intervention

Hula is the traditional dance of Hawai‘i and its indigenous people, with a history extending many centuries into the past [18, 19]. Although it is often misperceived solely as an entertainment for tourists, hula is the most popular of all Native Hawaiian cultural practices, and is performed by men and women of all ages. Originally performed to convey historical events and connections to the natural world, hula is now practiced as a form of cultural and creative expression. It reconnects Native Hawaiians with their native language, history, and values, and functions as a form of cultural identification. *Kumu hula* (hula masters) are the guardians and educators of the hula tradition and are recognized as cultural experts. At present, 193 *hālau hula* (hula schools) operate in Hawai‘i, while 1100 schools of dance teach hula worldwide [20]. Hula festivals throughout Hawai‘i and the continental U.S. draw thousands of dancers and supporters each year.

Hula comprises rhythmic body movements that illustrate the poetry of accompanying songs or chants [18, 19]. Movements can vary in intensity and duration, depending on the choreography, and can be modified for people with limited physical capacity. Several features make hula a viable strategy to prevent CVD among Native Hawaiians with HTN. Specifically, the energy expenditure of hula yields a metabolic equivalent (MET) of 5.7 for moderate-intensity and 7.6 for high-intensity forms [21]. Physical activity resulting in 3–6 MET (corresponding to an expenditure of 3.5–7 kcal/min) is considered moderate, while >6 MET (expenditure greater than 7 kcal/min) is vigorous [22]. In addition, the use of instrumental music and chanting, along with the discipline of coordinating complex body movements, is likely to reduce psychological stress and improve self-regulatory ability [23–25]. In traditional terms, hula represents a mind-body approach that emphasizes the value of *pono* (harmony) and spiritually that connects the dancer to the place, person, and event conveyed by the dance in an expression of *lōkahi* (unity).

### Physical activity interventions for hypertension

Physical activity is a major part of lifestyle interventions to reduce HTN and other CVD risk factors [26, 27]. In people with HTN, such interventions can lead to reductions in SBP of 5–10 mmHg and reductions in DBP of 1–6 mmHg [28] – comparable to the effects of sodium reduction and weight loss [26, 29, 30]. The positive effects of physical activity on HTN can be magnified when activity is coupled with other lifestyle changes (e.g., sodium reduction) or pharmacologic therapies [31]. Physical activity is a primary CVD prevention strategy endorsed by the American Heart Association, with recommendations for at least 30 min of moderate-intensity physical activity (40–60% of maximum heart rate capacity) on most days of the week [32, 33]. HTN interventions based on physical activity have demonstrated effectiveness [28, 34–36]. For example, *Taiji Quan*, a mind-body regimen originating in Chinese martial arts, has been used successfully for BP control [37]. In people with HTN, it has been shown to reduce SBP by 9.3–14.3 mmHg and DPB by 6.0–7.2 mmHg [38]. Other traditional forms of physical activity, such as hula, may have similar effects.

Many Native Hawaiians at risk for CVD find it difficult to engage in regular physical activity because of economic challenges and residence in obesogenic environments with few opportunities to engage in safe physical activity or eat a healthy diet [16, 39]. They find standard physical activity recommendations, such as biking or using treadmills, unappealing because they are individually-focused and discordant with cultural expectations [16, 40, 41]. Instead, they prefer physical activity that is consistent with their traditional values and modes of living, such as group-based programs that promote mind-body connections, spirituality, and cultural traditions [15, 40, 41]. Thus, we sought to provide a viable alternative to previous interventions by establishing an accessible, sustainable, and culturally-relevant HTN intervention for Native Hawaiians [10, 16].

### Pilot study and preliminary findings

We conducted a pilot randomized controlled trial (RCT) with a wait-list control group to test the effects of a 3-month hula-based intervention on reducing SBP in 55 Native Hawaiians and other Pacific Islanders with physician-diagnosed HTN and SBP >140 mmHg (or >130 mmHg for those with comorbid diabetes) [42]. The intervention was called *Ola Hou i ka Hula* (Restoring Health Through Hula), and it involved 2 60-min hula lessons per week delivered over 12 weeks, along with 6 30-min sessions on HTN self-management delivered between the hula lessons. Each hula group consisted of 12–15 participants. At 3-month follow-up, 72% of intervention paticipants demonstrated ≥10 mmHg reduction in SBP, versus 39% in the wait-list control group. The mean SBP reduction was −18.3 mmHg for the intervention group versus −7.6 mmHg for controls. The intervention group also reported significantly more reductions in bodily pain and improvements in social functioning, along with a reduction in perceived ethnic discrimination [42]. However, at 6-month follow-up (3 months after cesssation of the intervention), SBP in the original intervention group began to return to baseline.

### Definitively testing the effects of a hula-based intervention

With funding from the National Heart, Lung, and Blood Institute of the National Institutes of Health, we established the Kā-HOLO^1^ Project, a community-based participatory research (CBPR) project, to enhance the *Ola Hou i ka Hula* intervention (hereafter *Ola Hou*) by including an additional 3-month maintenance component. This component was informed by the self-regulation theory of behavior change [43], which emphasizes goal adoption for self-directed change, implementation of productive actions to achieve goals, and maintenance strategies to promote sustainable change by developing behavioral capability, self-control, and self-efficacy [44]. This approach has been successfully applied to HTN control in other populations [45, 46]. We then sought to perform a more definitive study of the effect of the intervention on HTN and CVD risk with a larger sample of Native Hawaiians. We developed a study protocol to address these 3 aims:

1) Test the efficacy of a 6-month hula intervention designed to reduce SBP in Native Hawaiians diagnosed with HTN by conducting an RCT with a wait-list control arm. *We predict that individuals in the intervention arm will have greater reductions in their SBP post-intervention compared with persons randomized to wait-list control, and will maintain these improvements at 12-month follow-up.*

2) Compare CVD risk scores in the intervention and wait-list control arms post-intervention and at 12-month follow-up. *We predict that NH randomized to the intervention will show greater improvements post-intervention in their 10-year CVD risk profile than those randomized to a wait-list control, and will maintain these improvements at 12-month follow-up.*

3) Test whether intervention effects are mediated through psychosocial and cultural factors by using structural equation modeling. *We predict that changes in these constructs will partially mediate the effect of the intervention on blood pressure reduction and CVD risk score, though a direct effect is also possible.*

The present article details the design and methodology of our ongoing intervention study. Subsequent publications will report the results.

## Methods

### Study design

**Fig. 1:**
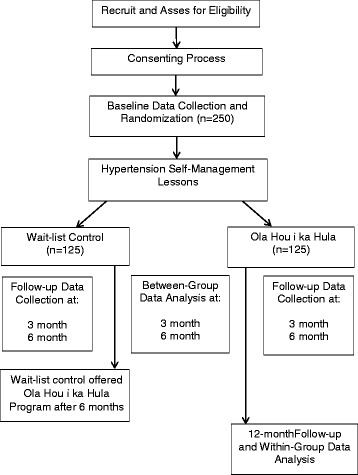
CONSORT Flowchart of Overall Study Design

An overview of the study design appears in Fig. 1. We are conducting a 2-arm RCT using a wait-list control. We will recruit 250 adult Native Hawaiians with confirmed HTN and perform a 1:1 randomization within each study site, such that 125 are assigned to the intervention and 125 are assigned to the wait-list control arm. The hula intervention will be delivered by experienced *kumu hula* and trained community peer educators to groups of 12 to 15 at each study site following standardized protocols. Our primary outcomes are reductions in SBP and improvements in CVD risk as measured by the Framingham Risk Score (FRS). We elected to focus on SBP instead of DBP because the former is a stronger predictor of CVD risk, and its predictive strength increases with age [47–49]. Changes in SBP and FRS from baseline will be compared between study arms at 3-month and 6-month follow-ups. Wait-list participants will be offered the hula intervention after completing their 6-month follow-up. The original intervention arm will also complete a 12-month follow-up to assess the maintenance of SBP and FRS improvements.

### Community-based participatory research approach

The present RCT is embedded in the KāHOLO Project, a larger 6-year project designed to address CVD in Native Hawaiians by using community-based participatory research and culturally-based intervention strategies. The initial design of the *Ola Hou* program was based on extensive consultation with *kumu hula* in order to maintain the cultural integrity of hula as a health-promoting intervention [50]. Input was also sought from patients with HTN and CVD to inform the development and refinement of the hula intervention and to further ensure its relevance and acceptability [40, 41]. A prominent *kumu hula* serves as the primary cultural advisor and trainer for *kumu hula* in the *Ola Hou* program at each study site to ensure intervention fidelity across sites. This study is also guided by a community advisory board composed of a *kumu hula* and 3 nationally-recognized experts in health promotion with indigenous populations. In accordance with the principles of community-based participatory research, as articulated by Israel et al. [51], a staff member at each study site serves as a community investigator.

Six community-based organizations (CBO) and two academic institutions are part of the Kā-HOLO Project: **Hālau Mōhala ‘Ilima** is a Native Hawaiian cultural education school with several hundred students aged 5–90 years; it is the training site for *kumu hula* with regard to cultural and research protocols. **Kula no nā Po‘e** is a nonprofit community center serving the educational and health needs of residents of Native Hawaiian homestead communities in urban center of Honolulu. **Ke Ola Mamo** is the Native Hawaiian Health Care System for the island of O‘ahu. **Wai‘anae Coast Comprehensive Health Center** is a federally-qualified community health center located in a rural area of O‘ahu. **Hui no ke Ola Pono** is the Native Hawaiian Health Care System for the island of Maui. **I Ola Lāhui: Hawai‘i Rural Behavioral Health Program** is a nonprofit behavioral health training program that cares for underserved and Native Hawaiian communities. **Initiative for Research and Education to Advance Community Health (IREACH)** at Washington State University is an academic research center focusing on the health and well-being of American Indians, Alaska Natives, and other health disparities populations. **Department of Native Hawaiian Health (DNHH)** is a clinical department in the John A. Burns School of Medicine at the University of Hawai‘i focusing on the health and well-being of Native Hawaiians, Pacific Islanders, and other health disparate populations.

### Procedure

#### Recruitment

Native Hawaiian participants will be recruited by 5 of our CBO partners at their respective sites through existing client or patient registries or regular community events (e.g., health screenings and community programs). About 20 to 40 participants will be recruited at each site by a community-based researcher trained in research ethics. All sites have experience with recruitment for RCTs or health promotion interventions, and all have access to a large enough pool of Native Hawaiians to recruit a sufficient number of participants with HTN over a 3-year accrual period.

#### Inclusion and exclusion criteria

Inclusion criteria include age > 20 years; Native Hawaiian ancestry; physician-diagnosed HTN and continued indications of SBP >140 mmHg (or SBP >130 mmHg with diabetes); physician’s approval to participate in moderate physical activity (e.g., 120 min of hula per week); and no prior history of CVD. The only exclusion criterion is pregnancy. Patients taking HTN medication, regardless of prescribed regimen, will be allowed to participate. All participants will be instructed to continue their usual medical care, and all will be made aware that participating in the study does not replace their usual care or their providers’ medical recommendations.

#### Informed consent

For those eligible and willing to participate, informed consent will be obtained by a trained community-based researcher at each participating site. All participants will be informed of the intent of the study, the assessment measures, the randomization process, and the potential risks and benefits. The institutional review boards of the University of Hawai’i and Washington State University have approved all study procedures.

#### Baseline and follow-up assessments

**Fig. 2:**
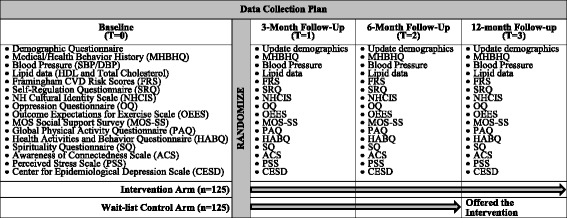
Overall Timeline for Baseline and Follow-up Data Collection

Figure 2 summarizes the overall timeline for baseline and follow-up data collection. We will collect data on demographics and medical history from each participant at baseline. Demographic data include 1) date of birth, 2) gender, 3) marital status, 4) highest educational attainment, 5) ethnic ancestry, and 6) past experience with hula. Medical and health behavior data include 1) smoking history and status, 2) frequency and intensity of alcohol use, 3) family history of CVD and diabetes, 4) CVD risk factors (e.g., history of high cholesterol, HTN, and diabetes), and 5) prescribed HTN medication, including type, brand, and dose. Medication adherence will also be assessed with a brief survey [52]. Weight will be measured by an electronic scale (Tanita BWB800AS). We anticipate that randomization will ensure a balance in demographic and medical characteristics across study arms. In the unlikely event that subsequent analyses reveal unbalanced samples, these baseline data will help to statistically control for potential confounders.

We will measure each participant’s SBP and DBP in mmHg at baseline with an automatic BP machine (Omron©HEM-907XL, Omron Healthcare) [53–55]. Standardized protocols for obtaining BP will include taking 3 separate BP measures during the baseline visit and averaging the last 2 to calculate mean SBP and DBP for each participant [56].

To evaluate CVD, we will collect blood cholesterol data needed to calculate participants’ FRS by using the Alere Cholestech LDX® System. FRS is an algorithm that estimates a person’s 10-year risk of developing CVD [57]. It has been used to estimate change in CVD risk status in previous lifestyle interventions conducted with underserved populations with HTN [58, 59]. To calculate FRS, a drop of capillary blood is drawn from each participant by fingerstick, the least invasive means of obtaining lipid data [60]. Data on HDL and total cholesterol, along with age, gender, smoking status, and SBP, will be entered in an online FRS calculator to obtain a 10-year CVD risk score for each participant, with lower scores indicating lower risk.

To measure potential mediators of the intervention effect, we will implement the following measures, all of which are hypothesized to affect BP: 1) the Self-Regulation Questionnaire to assess behavioral and emotional self-control in planning, delay of gratification, self-reinforcement, problem-solving/coping, and negative emotion regulation [61, 62]; 2) the Native Hawaiian Cultural Identity Scale to assess degree of identity and affiliation with cultural group and the impact of cultural group on lifestyle [63]; 3) a 5-item version of the Oppression Questionnaire to assess perceived ethnic discrimination [63, 64]; 4) the Global Physical Activity Questionnaire to assess time spent in moderate, vigorous, and sedentary activity [65]; 5) the 9-item Outcome Expectations for Exercise Scale to assess perceived benefits of exercising [66, 67]; 6) the Medical Outcomes Study Social Support Survey to assess emotional and informational supports [68]; 7) a Health Activities and Behaviors Questionnaire to assess health behaviors, including smoking and alcohol; 8) a 7-item spirituality questionnaire to assess spirituality and connection to a higher power [69]; 9) the 12-item Awareness of Connectedness Scale to assess feelings of connection with the environment, family, and community [70]; 10) the 10-item Perceived Stress Scale to measure psychological stress [71]; 11) the 10-item Center for Epidemiological Studies Depression Scale [72]; and 12) a 4-item survey to evaluate medication adherence [52].

To more objectively evaluate physical activity, a Fitbit Flex (Fitbit©, San Francisco) will be given to participants to wear for 1 week at baseline, 6-month follow-up, and 12-month follow-up (only for the original intervention arm). The Fitbit Flex is a small (113 g) accelerometer-based activity monitor worn on the wrist to track the daily number of steps taken, distance travelled, calories burned, and minutes of moderate physical activity. It connects wirelessly to a computer or smartphone to display the data collected. As an incentive, participants will be able to keep the Fitbits at the end of the study.

For all follow-up assessments, the same measures administered at baseline will be re-administered, with the exception of the sociodemographic measures, along with the mediator measures. At each visit, these measures will require 15 to 35 min to complete. For participants randomized to the intervention arm, follow-up data collection will happen at 3 months, 6 months, and 12 months. Collecting data at 3 months will allow us to compare the effects of the previously tested 3-month component against its effects in the ongoing RCT, while the 6-month follow-up will assess the effects of the add-on component intended to guide behavior change. The 12-month follow-up will assess post-intervention maintenance of SBP control. Participants in the wait-list control condition will contribute follow-up data only at 3 and 6 months. For control participants, the 3-month follow-up was designed to reduce attrition by keeping them engaged and providing an additional incentive to remain in the study. The efficacy of the hula intervention will be assessed by comparing baseline, 3-month, and 6-month data between intervention and control participants.

#### Randomization

Our choice of 1:1 randomization at each study site will reduce the effect of potential confounders unique to each site. An ordered list of study group assignments stratified by site will be generated (i.e., computer-generated random numbers) to maximize balance. To ensure unbiased randomization, study assignments will be made before baseline data collection, but participants and study staff at community sites alike will be blinded to participant assignments until baseline data collection is complete. At each site, randomization will be performed in cohorts of 20 to 40 participants each, with half randomized to the intervention and the other half to the control condition. Over the 3-year accrual period, each study site will recruit at least one cohort of 20 participants per year. Randomization will be conducted by a DNHH study staff without any input from the academic and community investigators.

#### Hypertension (HTN) self-management course

To assess the unique effects of the hula intervention on HTN control, above and beyond HTN education, all participants will complete a 3-h HTN self-management course before receiving the hula lessons or entering waitlist condition. This course includes 3 lessons: 1) Hypertension 101 (signs and symptoms of HTN and physical activity), 2) Medicines, and 3) What’s in your food? (i.e., heart-healthy eating, including sodium reduction). Each lesson is delivered by a community peer educator and lasts 1 h, with all lessons delivered over a 1-week period. An additional lesson on managing negative emotions will be delivered during the maintenance phase of the intervention only to intervention participants as part of the self-regulation focus.

#### Intervention arm

Participants randomized to the intervention will begin the 6-month *Ola Hou* program within 1 week of completing the HTN self-management course. The first 3 months of the intervention were designed and standardized as a culturally-based program of physical activity involving 12 weeks of hula training. Training will be delivered in the form of two 60-min classes per week over 12 weeks (24 classes total). Each class will include about 10 to 20 participants, and will offer opportunities for social engagement and support. The hula class format in this study was previously evaluated and found to offer an appropriate level of physical activity in accordance with national recommendations [21], including recommendations for people with limited mobility and fitness [41]. Lessons are led by a *kumu hula* trained in the standardized protocols developed by the study’s lead *kumu hula*. Each 60-min lesson aims to increase the amount of time engaged in continuous dancing, from 5 to 10 min in week 1 to 40 min by week 12. BP devices will be given to participants to help them regularly self-monitor BP throughout the study period. Table 1 outlines the phases, activities, and goals of each lesson.

**Table 1:** Summary of each 60-min hula lesson in the *Ola Hou* program

Phase	Duration	Activities and goals
Warm-up	5–15 min	Stretching legs, arms, and lower back Low-level hula activity at 25–40% MPHR
Conditioning	20–40 min	Moderate-intensity hula 40–85% VO_max_ or 50–70% MPHR Training intensity = (40 + [2 x Max MET]) % RPE = Between 12 and 16 on Borg scale^a^ Target HR = (HR reserve x training intensity [%]) + HR_resting_
Cool-down	3–10 min	Low-level hula to return HR and blood pressure to resting level

*Abbreviations: MPHR* maximum predicted heart rate, *VO*_*max*_ maximal oxygen consumption, *MET* metabolic equivalent, *RPE* rating of preceived exertion, *HR* heart rate

^a^The Borg scale is a subjective measure of physical exertion. Scores range from 0 to 20

Hula styles, choreography, songs, and chants similar to those used in the pilot trial of the *Ola Hou* program will be used in the ongoing study. Aside from dancing, the *kumu hula* will teach the cultural meaning of each hula dance, its movements and gestures, and the words of the accompanying song or chant. Each hula tells a story of a specific location in Hawai‘i and its historical and cultural significance, emphasizing its relevance to Native Hawaiian values of *mālama ‘āina* (land stewardship), *lōkahi* (living harmoniously with others and surroundings), *hana pono* (socially appropriate behaviors), and *aloha* (compassion).

For the remaining 3 months of the *Ola Hou* program, a 60-min hula lesson will be offered only once per month to practice previously learned hula dances and learn a new hula dance with the same physical activity goals as previous lessons. In addition, the intervention arm will meet with the community peer educator each week during this period. Each meeting will last 45 min and will cover such self-regulation strategies as setting physical activity goals, self-monitoring physical activity and BP every day, and using self-monitoring data to modify physical activity goals. These group meetings will offer an opportunity for participants to set individual goals for physical activity and to practice hula while planning to engage in other forms of exercise to encourage the sustained adoption of new health behaviors. Developing self-regulation skills specific to HTN is assumed to increase the likelihood of sustained positive behavioral change.

As noted above, months 4 through 6 of the hula intervention were added to the original design after pilot testing suggested that this change would improve long-term maintenance of SBP improvements achieved during the first 3 months.

#### Wait-list control arm

After completing the 3-h HTN self-management course, participants randomized to the wait-list control arm must wait until they finish the 6-month data collection before they begin the *Ola Hou* program. In the meantime, they will be instructed to continue their usual medical care, but they will be neither encouraged nor restricted from participating in any other activity or intervention program while they wait.

#### Retention and incentive plan

All participants will receive a gift card incentive equivalent to $25 at each data collection visit, with total incentives of $100 for completing all 4 visits. For the wait-list control arm, the fourth payment will be an incentive to start the *Ola Hou* program at month 12. This strategy will ensure that all participants receive the same dollar amount of incentives, thereby avoiding any perception of unfairness – an important consideration in close-knit Native Hawaiian communities. Intervention participants will also receive an ambulatory upper-arm band BP device (valued at $40) to monitor their progress, which they can keep after the study is completed. Wait-list control participants will receive the same device when they begin the intervention. Reminder calls or emails will be made to both intervention and control participants 1 week before each data collection visit, and follow-up calls or emails will be made to allow participants who miss a data collection visit to reschedule. Participants who miss a visit and do not reschedule will be considered dropouts, and study staff will attempt to re-engage them. In our pilot study, these retention strategies successfully retained 87% of participants over a 3-month period. They should be similarly effective over the 12-month period required by the ongoing study [42].

#### Fidelity checks and data safety monitoring

To ensure the implementation fidelity of the hula intervention, research staff will intermittently visit hula classes and use a behavioral checklist to record the degree to which *kumu hula* and community peer educators follow standardized protocols. Booster training will be given as needed based on these checks. Participants will be tracked to record the number of hula and heart health classes they attend. In addition, research staff will assemble a data safety monitoring board (DSMB) composed of experts in cardiology, biostatistics, and clinical trials at study initiation who are independent from the investigators and sponsors and who will meet at least annually and as needed (e.g., review adverse events if any). The board will be responsible for reviewing trial data on an ongoing basis to ensure the safety of study participants.

#### Data management and data quality assurance

We will establish survey and data entry forms and a study databases using the research electronic data capture system (or REDCap), which allows secure and easy multi-site online access, mid-study modifications, and easy data tracking. REDCap is well-suited for CBPR that includes investigators working from different community sites. The staff of the Biostatistics and Data Management Core at the University of Hawai‘i will develop data collection instruments and a code with proper branching logic and stop actions. Participants’ data will be entered by study staff at each participating community site. A database manager will monitor data submitted online on an ongoing basis for quality control purpose and communicate with study staff to resolve any online database access and data entry issues in real-time. The REDCap database will be password protected and include the necessary encryption program to ensure data security with access only by trained research staff. All hardcopies of the participants questionnaires/surveys and lipid data will be kept in a locked, secure file cabinet in a locked room at the respective partnering community site, which will be accessible only to trained research staff. All research staff, both community and academic, will complete human subject in research training.

#### Protections against risk

One of the protection procedures for the participants is to obtain written approval by their personal physician before enrolling in the study to ensure they can participate in moderate physical activity safely. Having this approval will ensure they are also seen by their physician and that the physician is aware of participation in this study, providing the opportunity for a medical evaluation of his/her HTN (important for those who have not seen their physician within the past 6-months). A protocol will be established in the event that a participant’s blood pressure exceeds a safe level, systolic blood pressure > 180 mmHg or diastolic blood pressure > 110 mmHg, either during an assessment or during self-monitoring while in hula lesson. If BP exceeds the above cut off points, participants will wait 5 min and will be measured again. If BP is still elevated, they will be asked to go home and see their doctor as soon as possible. The participant will then be allowed to re-enter the study when cleared by their primary physician and no longer symptomatic; provided that this is done within 2 weeks. All adverse events will be immediately reported to the Principal Investigator and the DSMB for review and follow-up.

Trainings will be held for kumu hula and community peer educators, conducted by a clinician, to teach the symptoms and signs of physical distress, such as headache or blurred vision, confusion, chest pain, shortness of breath, dehydration, and edema. They will be taught how to handle these issues and when medical or emergency services are required. In the rare case that the intervention or data collection causes psychological distress, the Principle Investigator, who is a licensed clinical psychologist, will be consulted. He will meet with the participant to debrief and to determine if a referral to another mental health professional is necessary for further follow-up.

#### Statistical analysis

For our first two research aims, statistical analyses will be done on an intention-to-treat basis, with differences between study arms in mean changes in SBP and FRS as the primary outcomes. Participant characteristics and rates of recruitment, retention, and intervention fidelity will be summarized by using descriptive statistics. To test the hypothesis that the intervention will lead to significantly more reduction in SBP and FRS relative to the control condition, mean changes in SBP and FRS between baseline and 3-month and 6-month follow-up will be analyzed by independent t-tests between the 2 arms. Analysis of covariance and general linear modeling approaches will then be used to adjust for relevant covariates, such as gender, age, type of community, and baseline SBP and FRS values. Missing values for SBP and FRS at follow-up visits will be imputed by carrying forward baseline observations. If a covariate value is missing, it will be assumed to be missing at random. For sensitivity analyses, mean changes in SBP and FRS of participants who complete follow-up data collection will be analyzed by using a complete case analysis method. A stratified analysis will also be conducted within site or by gender to explore the potential impact of such factors on outcomes. In addition, data collected at all study visits will be modeled by repeated measures analysis of variance and linear mixed effects models, incorporating correlated data from multiple visits, to examine the post-intervention maintenance of SBP and FRS improvements. Determination of the appropriate covariance matrix in the repeated measure analysis will be based on quality of fit to the data by using the method of Fitzmaurice et al. [73]. Statistical analyses will be performed in SAS 9.4 (2015 SAS Institute Inc.). A two-tailed *p*-value <0.05 will be regarded as statistically significant.

To estimate whether the intervention effect is mediated by covariates, structural equation modeling will be performed by using Mplus [74], based on the following test for mediation: Intervention ➔ Mediator (path a), Mediator ➔ Outcome (path b). Intervention status (intervention vs. wait-list control) is a binary variable specified as exogenous (i.e., not predicted by other variables in the model). Covariates, including the Time 1 value of the mediator and potential confounders, can be specified along with intervention status, as appropriate. A postulated mediator is specified as predicted by the intervention effect (path a) and the BP outcome is predicted by paths from the mediator (path b) and a direct path from the intervention (path c). This yields coefficients for the 2 paths above and their standard errors, as well as an estimate of the direct effect (path c: intervention ➔ outcome).

The hypothesized mediation pattern is a significant path from the intervention to a given mediator (e.g., self-control), and a significant path from the mediator to a given outcome (e.g., SBP), indicating that at least part of the intervention effect is due to change in the mediator. The indirect effect is analyzed as the product of the two paths divided by an estimate of its standard error [75, 76]. Mediation can be tested for individual paths or a set of paths. Fritz and MacKinnon [77] have shown that the most powerful method to apply is the bias-corrected bootstrap test, available in Mplus. Simulations show that, with modest effect sizes (standardized coefficients of .20–.25) for path a and path b above, sample sizes in the range of 120–160 participants provide .80 power to detect an indirect effect that truly exists.

#### Power analysis and sample size

Our proposed target of 250 enrolled participants (125 per arm) is based on an anticipated attrition rate of 20%. To test the efficacy of the intervention, our sample size calculation was based on mean differences in SBP in our pilot study [42], which demonstrated a 3-month mean change in SBP of 7.5 mmHg (SD = 16.5) between 2 arms randomized 1:1. Assuming that 206 participants (103 per arm) complete all follow-up assessments, we will have 90% statistical power to detect a similar effect size in our ongoing study.

Although we plan to compare CVD scores between the tow study arms, no FRS data specific to the Native Hawaiian population are available. Therefore, we used data from a previous community-based intervention to calculate the necessary sample size. In a 1-year study with a multiethnic sample recruited in medically underserved urban and rural communities (*n* = 195; 59% White, 37% Black, and 4% Hispanic), the biggest changes in FRS were observed at month four [59]. The reported average reduction in FRS varied according to differences in initial risk profiles – for example, 6.0% (SD = 9.9%) for participants at high risk (FRS ≥ 20) and 1.3% (SD = 4.5%) for participants at intermediate risk (20 > FRS ≥ 10). Among study participants, 63% had HTN, with baseline averages of SBP = 146 mmHg and DBP = 82 mmHg, almost identical to the baseline averages in our pilot study (SBP = 146 mmHg, DBP = 86 mmHg). Given a similar mean change in FRS for participants at intermediate risk, a sample of *n* = 206 will provide 80% statistical power to detect a change in FRS of 1.3% (SD = 4.5%) at 6-month follow-up.

#### Resource sharing and dissemination plan

This study will include data from approximately 250 participants with hypertension, recruited over a period of 4 years. All investigators are aware of and agree to abide by the principles for sharing research resources, as described by the National Institutes of Health (NIH) in “Principles and Guidelines for Recipients of NIH Research Grants and Contracts on Obtaining and Disseminating Biomedical Research Programs." The final dataset will include self-reported demographic, survey, and clinical data.

Sharing of data generated by this project is an essential part of our proposed activities and will be carried out in several different ways. We will make our results available both to the community partners, community members, and scientists interested in our study. Our plan includes presentations at each community site, local and international cultural gatherings, as well as scientific conferences and meetings. We plan to publish our results in scientific peer-reviewed journals following the aforementioned presentations and approval from our community investigators. All publications generated from this project will include community members as contributing authors.

## Discussion

The principal strength of our ongoing intervention study is the use of hula, a popular cultural practice, as the basis of a program to prevent CVD in Native Hawaiians with HTN. Preliminary data strongly support this culturally sensitive approach. Our study is designed to link statistically significant changes in SBP with 10-year CVD risk, a clinically meaningful outcome. Provided the intervention has a significant positive effect, we will be able to explain how the *Ola Hou* program leads to reductions in SBP among Native Hawaiians through our analysis of important psychosocial and cultural mediators.

Our choice of an RCT with a wait-list control design ensures that we have a control condition to enable causal inference while providing all participants with an opportunity to receive the hula intervention. Although this experimental approach is not the most robust among RCT designs, it is acceptable to Native Hawaiians and has been used successfully in past studies with their communities [60]. We anticipate that our study findings will inform future investigations of other traditional cultural dances. Such investigations can evaluate their promise as intervention strategies for reducing CVD risk in indigenous populations in the U.S., Canada, New Zealand, and Australia.

Our study introduces several innovations. Using hula to prevent CVD is a novel approach in the context of contemporary prevention paradigms. Current strategies to prevent CVD are not consistent with Native Hawaiian values and preferences, because they focus on the individual, provide physical activity regimens that are irrelevant to indigenous culture, and are difficult for Native Hawaiians to access or sustain. Instead of culturally adapting an existing Western, evidence-based intervention, we chose a popular traditional Native Hawaiian practice as the basis for our program. The popularity and cultural relevance of hula mean that the *Ola Hou* program can be easily adopted and sustained in any community where Native Hawaiians live, whether in Hawai‘i or elsewhere.

Our outcome analyses will also include FRS, which have strong predictive power for 10-year CVD risk [78, 79]. These risk scores will include data on SBP and other major determinants of CVD risk, including lipid levels, smoking status, gender, and age [57]. These data will enable us to ascertain the longer-term clinical significance of our intervention within our 5-year study period.

Another innovation of this study is our analysis of psychosocial and sociocultural constructs (e.g., cultural identity, perceived ethnic discrimination) that might mediate the intervention effect on BP control. Our pilot study found that the intervention reduced perceptions of racism, and preliminary findings of other ongoing studies indicate that Native Hawaiian ethnic identity is positively related to behavioral and emotional self-control. Thus, our design responds to a transdisciplinary call for studies of the mechanisms underlying behavioral interventions [80–82].

Our ongoing study also has limitations. First, implementing the intervention at several different sites makes it difficult to ensure the consistency in delivery required by standardization, and might therefore introduce confounders into our analyses. To minimize this possibility, we will conduct fidelity checks and offer booster training sessions as needed. Second, we rely on self-report measures for many variables of interest, potentially introducing biases involving recall and social desirability. Nevertheless, self-report is the primary method of assessing attitudes, beliefs, and other cognitive processes, and we will use measures validated in previous studies with Native Hawaiians to minimize self-reporting biases. Third, we anticipate that more women than men might participate, since dancing might be more attractive to women. Accordingly, we will make special efforts to recruit Native Hawaiian men to achieve balanced gender representation.

## Abbreviations

BP: Blood pressure
CVD: Cardiovascular disease
DBP: Diastolic blood pressure
FRS: Framingham Risk Score
HTN: Hypertension
RCT: Randomized controlled trial
SBP: Systolic blood pressure

## Footnotes

1 Kāholo is the Hawaiian name of a common hula dance movement but HOLO here is also the acronym for Hula Optimizing Lifestyle Options.
